# Phylogeography and molecular diversity of two highly abundant *Themisto* amphipod species in a rapidly changing Arctic Ocean

**DOI:** 10.1002/ece3.10359

**Published:** 2023-07-30

**Authors:** Ayla Murray, Kim Præbel, Andrea Desiderato, Holger Auel, Charlotte Havermans

**Affiliations:** ^1^ Helmholtz Young Investigator Group ARJEL – Arctic Jellies, Functional Ecology Alfred Wegener Institute Helmholtz Centre for Polar and Marine Research Bremerhaven Germany; ^2^ BreMarE – Bremen Marine Ecology, Marine Zoology Universität Bremen Bremen Germany; ^3^ Norwegian College of Fishery Science UiT The Arctic University of Norway Tromsø Norway; ^4^ Department of Invertebrate Zoology and Hydrobiology University of Lodz Lodz Poland

**Keywords:** genetic diversity, hyperiid, mtCOI gene, population genetics

## Abstract

Rapid warming in the Arctic is drastically impacting marine ecosystems, affecting species communities and food‐web structure. Pelagic *Themisto* amphipods are a major component of the Arctic zooplankton community and represent a key link between secondary producers and marine vertebrates at higher trophic levels. Two co‐existing species dominate in the region: the larger *Themisto libellula*, considered a true polar species and the smaller *Themisto abyssorum*, a sub‐Arctic, boreal‐Atlantic species. Recent changes in abundance and distribution ranges have been detected in both species, likely due to the Atlantification of the Arctic. The ecology and genetic structure of these species are understudied, despite their high biomass and importance in the food web. For both species, we assessed genetic diversity, patterns of spatial genetic structure and demographic history using samples from the Greenland shelf, Fram Strait and Svalbard. This was achieved by analysing variation on the mitochondrial cytochrome c oxidase subunit 1 gene (mtCOI). The results revealed contrasting levels of mtCOI diversity: low levels in *T. libellula* and high levels in *T. abyssorum*. A lack of spatial genetic structure and a high degree of genetic connectivity were detected in both species in the study region. These patterns of diversity are potentially linked to the impacts of the Last Glacial Maximum. *T. libellula* populations may have been isolated in glacial refugia, undergoing gene flow restriction and vicariant effects, followed by a population expansion after deglaciation. Whereas *T. abyssorum* likely maintained a stable, widely distributed metapopulation further south, explaining the high diversity and connectivity. This study provides new data on the phylogeography of two ecologically important species, which can contribute to predicting how zooplankton communities and food‐web structure will manifest in the rapidly changing Arctic.

## Graphical abstract

This study investigates the genetic diversity, connectivity and population structure of the two abundant pelagic amphipod species *Themisto libellula* (Figure a) and *Themisto abyssorum* (Figure b) in the Arctic. This was achieved using DNA sequence variation at the mitochondrial cytochrome *c* oxidase subunit 1 gene. We show, for the first time, contrasting molecular diversity between the two congeneric species, as well as a lack of genetic structure in both species.

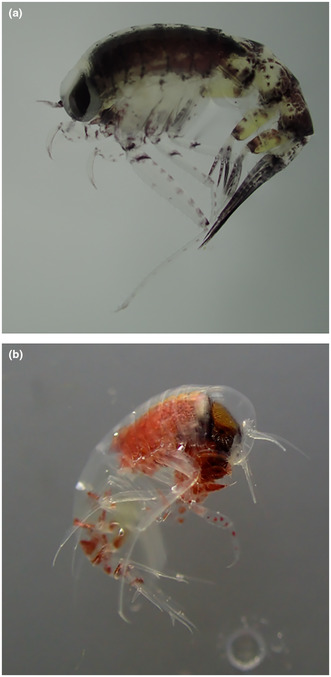

## 1 INTRODUCTION

The Arctic is currently warming three to four times faster than the global mean, and many consequences of climate change have now manifested throughout the region (Pörtner et al., 2019; Rantanen et al., 2022). Ocean and air temperatures are increasing rapidly, sea ice coverage and thickness are declining, and glaciers are melting region wide (Stroeve et al., 2012; Wang et al., 2020). These environmental changes are already having drastic impacts on the marine ecosystem, affecting species composition, distribution and food‐web structure in the Arctic Ocean (Gluchowska et al., 2017; Weydmann et al., 2014).

**FIGURE 1:**
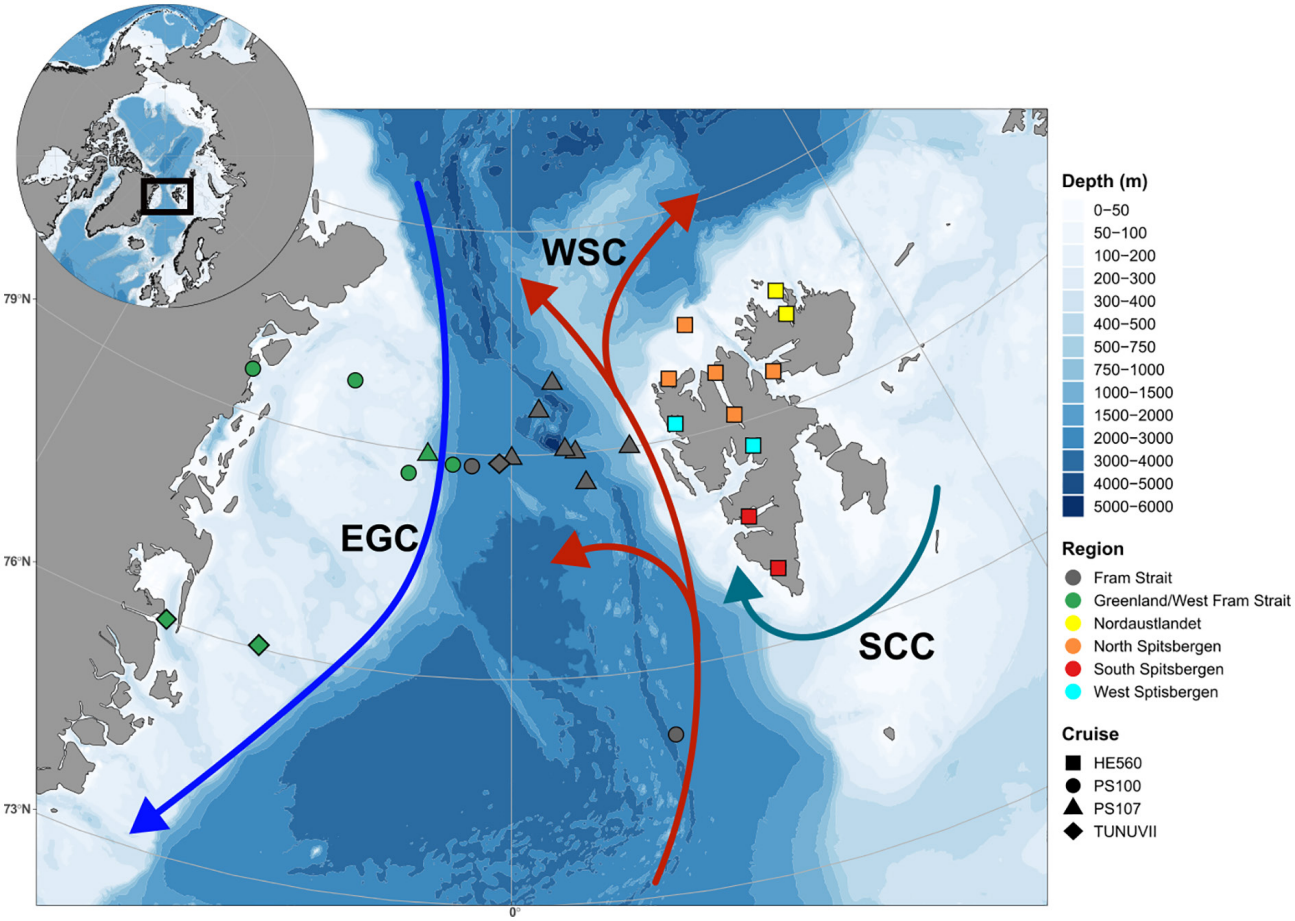
Map of study area including the Greenland Shelf, the Fram Strait and the Svalbard Archipelago. Sampling stations are indicated by points. Point shape indicates oceanographic cruise and point colour indicates major geographical regions. Major ocean currents are represented by arrows: colder currents in blue and warmer in red. EGC, East Greenland Current; SCC, South Cape Current; WSC, West Spitsbergen Current.

The inflow of increasingly warmer Atlantic water into the high Arctic is a major driver of the phenomenon commonly referred to as the ‘Atlantification’ of the Arctic (Polyakov et al., 2017). The Fram Strait, between Greenland and the Svalbard Archipelago, is known as ‘the gateway to the Arctic’ and is the largest source of oceanic heat into the Arctic Basin (Beszczynska‐Moeller et al., 2011). The heat exchange between water masses in the Fram Strait is influenced by two opposing currents: the north‐bound West Spitsbergen Current (WSC) and the south‐bound East Greenland Current (EGC) (Figure 1). The WSC carries warmer and more saline Atlantic water northwards into the Arctic Basin, while the ECG carries cold, fresher water and sea ice southwards through the Fram Strait (Figure 1) (Wang et al., 2019).

The WSC transports increasing amounts of nutrients and subarctic and boreal‐Atlantic plankton species into the Arctic (Csapó et al., 2021; Descôteaux et al., 2021; Gluchowska et al., 2017; Kraft et al., 2013), the consequences of which are not yet widely studied. Zooplankton community composition and functional changes have been detected in the region and linked to this Atlantification (Gluchowska et al., 2017). Poleward range expansions of subarctic and boreal‐Atlantic zooplankton species, as well as poleward contractions of polar species have also been observed (Andrews et al., 2019; Basedow et al., 2018; Csapó et al., 2021; Dalpadado et al., 2016). Environmental changes will continue to alter the distribution of suitable habitats and are expected to have an impact on gene flow and genetic structure in a wide range of zooplankton species in the Arctic and its marginal seas (Hardy et al., 2011; Tempestini et al., 2020).

Pelagic amphipods, and Hyperiidea in particular, are dominant planktonic crustaceans in terms of biomass in the polar regions and are a crucial component of the Arctic food web (Vinogradov et al., 1996). They are an important link between secondary producers (mesoplanktonic grazers) and marine vertebrates at higher trophic levels. Their role as prey in the Arctic zooplankton community has been described as on par with that of krill and copepods (Bowman, 1960; Dalpadado, 2002). The predominant hyperiids in the Arctic Ocean are of the genus *Themisto* Guérin, 1825. Two coexisting species dominate: *Themisto libellula* (Lichtenstein in Mandt, 1822), considered a genuine polar species and *Themisto abyssorum* (Boeck, 1871), a subarctic, boreal‐Atlantic species. An invasive third species from the North Atlantic, *Themisto compressa* (Goes, 1865), has also been reported in the Fram Strait, albeit in lower abundances (Kraft et al., 2013). Both *T. libellula* and *T. abyssorum* are preyed upon by fish, seabirds and marine mammals throughout the Arctic (reviewed in Havermans, Auel, et al., 2019). They are visual predators of meso‐ and microzooplankton and although their geographical distributions overlap, they occupy different ecological niches (Auel et al., 2002).

**FIGURE 2:**
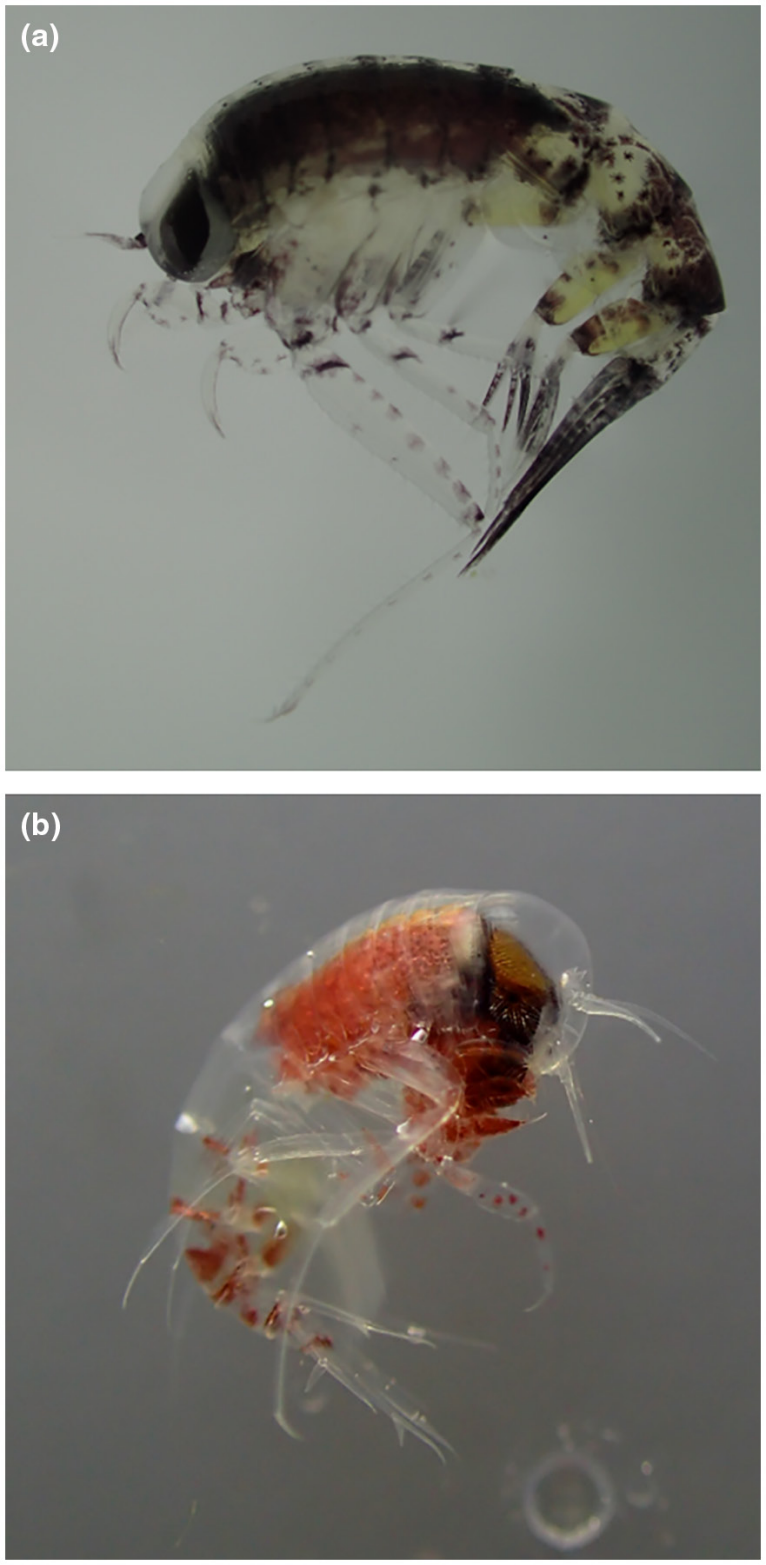
(a) *Themisto libellula* and (b) *Themisto abyssorum*. Size of the adult individuals typically ranged from 0.6 to 2.5 cm for *T. libellula* and from 0.4 to 1.3 cm for *T. abyssorum* in the study area (C. Havermans, personal observation).

*Themisto libellula* (Figure 2a) is a cold‐adapted species and its distributional range includes the Central Arctic Basin as well as its marginal seas (Havermans, Auel, et al., 2019). It can grow up to 60 mm in size and has a life cycle of up to 4 years (Auel & Werner, 2003; Kraft, 2010). An important component of its diet are ice‐dependent, herbivorous copepods, indicating a reliance on the cryo‐pelagic pathway (Auel & Werner, 2003; Kohlbach et al., 2016). It is a key prey item for seabirds, including the little auk (*Alle alle* (Linnaeus, 1758)), Arctic fish species such as polar cod (*Boreogadus saida* (Lepechin, 1774)) and commercially important fish such as Atlantic cod (*Gadus morhua* Linnaeus, 1758) and salmonid species (reviewed in Havermans, Auel, et al., 2019; Pinchuk et al., 2013).

*Themisto abyssorum* (Figure 2b) is largely found in waters of Atlantic origin (Mumm et al., 1998) in the marginal Arctic seas and has a life cycle of up to 2 years (Havermans, Auel, et al., 2019). It is smaller than *T. libellula*, and while lipid content by body mass is comparable, its smaller body size makes it an overall less nutritious prey (Auel et al., 2002). Previous studies indicate that the diet of *T. abyssorum* is less sea ice‐dependent than that of *T. libellula*, with a wider prey spectrum and a higher trophic position in the Arctic food web (Auel et al., 2002; Kohlbach et al., 2016).

Both species have exhibited recent changes in abundance and distribution, likely as a result of the Atlantification of the Arctic. *Themisto libellula* has decreased in abundances, whereas *T. abyssorum* has become more abundant in the Fram Strait and the Barents Sea (CAFF, 2017; Havermans, Auel, et al., 2019). These changes in abundance and distribution could have strong implications at higher trophic levels, with the loss of the highly nutritious *T. libellula* as a key prey item for many species (reviewed in Havermans, Auel, et al., 2019).

Many aspects of the current genetic diversity, population structure and phylogeography of *T. libellula* and *T. abyssorum* are not well studied, despite their importance in the Arctic food web and biogeochemical cycles (Havermans, Auel, et al., 2019). Investigating patterns of genetic diversity over a spatial distribution can give insight into how historic evolutionary events have impacted genetic variation in a species, as well as ongoing processes such as gene flow (Nei, 1987; Slatkin, 1987). The phylogenetic relationships between different *Themisto* species have been addressed (Tempestini et al., 2017), but our understanding of genetic diversity and distribution patterns are still limited within species, due to the lack of studies on small‐scale geographical structure. This study is the first, to our knowledge, to focus in depth on the genetic diversity and structure of *T. abyssorum*, and the first to compare these two congeneric species using molecular barcoding.

We applied the commonly used ‘barcode’ gene for animal taxa, a fragment of the mitochondrial cytochrome *c* oxidase subunit 1 gene (hereafter mtCOI) (Hebert et al., 2003), to characterize the phylogeography and molecular diversity of the two congeners *T. abyssorum* and *T. libellula*. We aimed to (i) assess and compare the levels of intra‐ and interspecific genetic diversity, (ii) determine present spatial patterns of genetic structure and connectivity, as well as (iii) provide an overview of the demographic history of the two *Themisto* species, across a broad geographical gradient in the Atlantic sector of the Arctic. This was achieved by analysing and comparing mtCOI gene variation according to the geographical location of samples from the Greenland shelf, the Fram Strait and the Svalbard Archipelago. Considering their relationship as congeneric species (Tempestini et al., 2017), similar life history patterns and dispersal capacities, we hypothesized comparative levels of genetic diversity at the mtCOI region. Both species have a holoplanktonic lifestyle, and considering the multidirectional currents and the lack of major geographical barriers in the study region, we predicted relatively high levels of genetic connectivity. As an alternative hypothesis, divergent genetic diversity patterns could be expected between the two species based on their current distributions and their link to distinct water masses (Dalpadado, 2002). From an eco‐evolutionary perspective, both species may have undergone different gene flow restrictions during the last glacial maximum (LGM) (Hardy et al., 2011). *Themisto libellula* may have had to survive the ice‐covered glacial periods in Arctic refugia, leading to diversification events after a bottleneck, whereas *T. abyssorum* may have been pushed south out of the Arctic, surviving as one large, connected population in ice‐free waters. Hence, alternatively, we expected potentially high levels of genetic divergence, with the possibility of cryptic species within *T. libellula*, and a higher genetic homogeneity in *T. abyssorum*.

## 2 MATERIALS AND METHODS

### 2.1 Study area

Samples were collected across the Greenland Shelf, the Fram Strait and the Svalbard Archipelago. The Greenland Shelf and the western Fram Strait are largely influenced by the ECG, whereas the central Fram Strait stations are deeper and influenced by the WSC. The West Spitsbergen stations are under the influence of the WSC, whereas the South Spitsbergen stations are affected by the South Cape Current (SCC). North Spitsbergen and Nordaustlandet stations are colder with less influence from the WSC (Figure 1).

### 2.2 Sample collection

Zooplankton samples containing *Themisto* amphipods were collected during oceanographic research cruises between 2016 and 2020 (Table 1 and Figure 1). Specimens from the Fram Strait and East Greenland were collected on the *R/V Polarstern* (Knust, 2017) cruises PS100 in 2016 (Kanzow, 2017) and PS107 in 2017 (Schewe, 2018), with a 150 μm Multinet and 300 and 500 μm Bongo nets. Multinet hauls were carried out vertically in the water column (0.5 m/s), Bongo nets were towed obliquely at 2 knots ship's speed, with a wire length varying between 20 and 450 m. Additional Fram Strait and East Greenland specimens were collected during the cruise TUNU‐VII in September 2017 on the *R/V Helmer Hanss*en, with a Campelen 1800 shrimp trawl (Walsh & McCallum, 1997). Specimens from the Svalbard Archipelago were collected in 2020 on the *R/V Heincke* cruise HE560 (Mark et al., 2022), using 300 μm and 500 μm Bongo nets and a pelagic trawl net fitted with a fish lift (Holst & McDonald, 2000). Samples were preserved immediately after collecting and sorting procedures in 96% undenatured ethanol. Six major geographical regions were defined based on ocean bathymetry and influences of major oceanic currents (Figure 2).

**TABLE 1:** Summary of samples sequenced: name and position of sampling stations, sampling gear and maximum depth of water column sampled and number of sequences obtained with 658 bp on mtCOI.

	Region	Sampling station	Expedition	Latitude	Longitude	Maximum sampling depth (m)	*T. libellula*	*T. abyssorum*
1	Fram Strait	2	PS100	75.11° N	8.54° E	345	0	22
2		87	PS100	79.79° N	11.89° W	250	2 (3)	0
3		102	PS100	78.84° N	2.74° W	345	7	3
4		2	PS107	78.59° N	5.05° E	345	24	18 (2)
5		21	PS107	78.96° N	0.02° E	191	24	0
6		38	PS107	79.01° N	4.49° E	345	24	0
7		32	PS107	79.59° N	2.03° E	345	14	0
8		34	PS107	79.95° N	3.13° E	345	17	0
9		45	PS107	79.00° N	8.31° E	345	0	17
10		7	PS107	79.06° N	3.77° E	345	8	0
11		1381	TUNUVII	78.86° N	0.63° W	26	5	0
12	Greenland/West Fram Strait	95	PS100	78.68° N	7.02° W	250	4 (2)	1
13		98	PS100	78.85° N	4.06° W	–	0	1
14		246	PS100	79.57° N	19.51° W	200	3	3
15		241	PS100	79.57° N	19.50° W	300	6 (3)	2
16		29	PS107	78.98° N	5.50° W	340	23	6
−17		1306	TUNUVII	75.99° N	19.46° W	480	7	0
18	1338	TUNUVII	76.01° N	14.19° W	350	10 (1)	0
19	Nordaustlandet	1	HE560	80.52° N	22.07° E	177	0	5
20		2	HE560	80.18° N	22.15° E	200	10	5
21	North Spitsbergen	4	HE560	79.52° N	19.67° E	200	10	12
22		5	HE560	79.74° N	15.54° E	123	0	7
23		6	HE560	79.13° N	16.02° E	169	9	15 (1)
24		9	HE560	80.46° N	14.19° E	50	10	0
25		10	HE560	79.81° N	12.00° E	146	0	12
26	West Spitsbergen	17	HE560	79.20° N	11.79° E	260	10	23 (2)
27		23	HE560	78.66° N	16.68° E	170	10	0
28	South Spitsbergen	24	HE560	77.76° N	15.14° E	100	10	0
29		25	HE560	76.99° N	16.01° E	100	3	0

*Note*: Numbers in parentheses () indicate shorter sequences (<658 bp) that were included in the genetic distance analysis and ML tree but removed from subsequent analysis.

### 2.3 DNA extraction, PCR and sequencing

For this phylogeographic study, DNA was extracted from 212 individuals of *T. abyssorum* and 281 individuals of *T. libellula*. Additionally, DNA was extracted from 23 *T. compressa*, for comparing interspecific variation between congeneric species. Between two and four pleopods were taken for DNA isolation from larger individuals, and the entire pleon including pleopods form the smaller individuals (<7 mm). DNA extraction was performed using the QIAGEN DNeasy Blood & Tissue Kit, in accordance with the manufacturer's instructions. Each DNA extraction was treated with 20 μL of proteinase K and only 100 μL of AE buffer was used for sample elution to ensure a higher concentration of DNA. DNA content was measured with a NanoDrop ND‐1000 (Thermo Fisher Scientific) and diluted to approximately 50 ng of DNA for subsequent polymerase chain reaction (PCR).

The target gene fragment of this study was the mtCOI barcoding region. This gene fragment has the advantage that it has low or no recombination, maternal inheritance and a faster evolutionary rate compared to nuclear DNA (Moritz et al., 1987). Diversity on mtCOI is higher within species than between species, making it a suitable tool for estimating intraspecific diversity in congeneric species (Meyer & Paulay, 2005; Vieira et al., 2022). Furthermore, population bottlenecks and expansions leave signatures in the mtCOI region, making it a popular tool for inferring elements of a species demographic history (Beermann et al., 2020; Hewitt, 2000). The 658 base pair (bp) barcoding region of the mtCOI was amplified using the universal ‘Folmer’ primers HCO 2198 (5′‐TAAACTTCAGGGTGACCAAAAAATCA‐3′) and LCO 1490 (5′‐GGTCAACAAAT‐CTAAAGATATTGG‐3′) (Folmer et al., 1994). The reaction mixture had a total volume of 25 μL and consisted of: 1x PCR buffer, 0.2 mM dNTPs, 0.5 μM forward and reverse primers, 0.2 U/μL HotMaster Taq DNA polymerase (QuantaBio), 1 μL (ca. 30 ng) of DNA template and molecular‐grade water. DNA template was substituted with molecular‐grade water in negative controls. The PCR amplification was carried out using the following programme: initial denaturation at 95°C for 2 min; followed by 36 cycles of denaturation at 94°C for 20s, annealing at 42°C for 20s, extension at 65°C plus a final extension at 65°C for 15 min. PCR products were checked for quality and length using electrophoresis on GelRed‐stained, 2% agarose gel and were bidirectionally sequenced by EUROFINS (Germany).

### 2.4 Sequence editing and alignment

Chromatograms were manually checked for ambiguous base calls and stop codons in amino acid translations, using the software CodonCode Aligner 8.0.2 (Codon Code Corporation). The primers were removed, and all sequences were trimmed to a maximum 658 bp length. Low‐quality sequences were removed, resulting in a total of 260 *T. libellula*, 157 *T. abyssorum* and 22 *T. compressa* sequences (Table 1). A consensus of all forward and reverse amplicons was created, and all sequences were aligned with CLUSTAL‐W (Thompson et al., 1994) in CodonCode Aligner. Morphological identification of all sequenced specimens was confirmed by the mtCOI barcoding and searched on the Basic Local Alignment Search Tool (BLAST) provided by National Center for Biotechnology Information (https://blast.ncbi.nlm.nih.gov/Blast.cgi). Specimen data and generated sequences were submitted to the Barcode of Life Data Systems repository (BOLD, Ratnasingham & Herbet, 2007), under the project name ‘ARCTH’ (BOLD ID: ARCTH001‐22 – ARCTH438‐22) and on GenBank (Accession numbers: OR210933 – OR211370).

### 2.5 Genetic diversity analysis and demographic history

Sequences were collapsed into unique haplotypes and used to construct a maximum likelihood (ML) phylogeny tree using PhyML (http://www.atgc‐montpellier.fr/phyml; Guindon et al., 2010). The best fitting substitution model (TN93 + G + I) was found according to both AIC and BIC values using SMS (Lefort et al., 2017). The software FigTree v1.4.4 (http://tree.bio.ed.ac.uk/software/figtree/) was used to edit the subsequent ML tree. Statistical support for the clades was estimated with 1000 bootstrap replicates.

The Kimura 2‐parameter (K2P) substitution model with pairwise deletion and 10,000 bootstrap replicates (Felsenstein, 1985) was used to calculate intra‐ and interspecific genetic distances on the full sequences (Kimura, 1980), using Mega 11 software (Kumar et al., 2018).

The *T. compressa* sequences (*N* = 22) were only included in the genetic distance analysis and the ML tree to illustrate the relationship between the three congeneric species but were not used further in this study. An additional 33 publicly available sequences of *T. libellula* from the Pacific sector of the Arctic (Tempestini et al., 2020) (GenBank accession numbers: MT832367–MT832393) were included in the genetic distance analysis and ML tree to investigate their relationship to the *T. libellula* in the present study, but not included in subsequent analysis. Furthermore, five sequences of the hyperiid *Hyperiella dilatata* Stebbing, 1888 from a previous study (Havermans, Hagen, et al., 2019) were added to the tree as an outgroup (GenBank accession numbers: MH482519–MG482623). For all subsequent analyses, sequences of different fragment lengths were excluded to avoid base‐pair loss, resulting in a final dataset of 250 *T. libellula* and 152 *T. abyssorum* sequences.

Intraspecific genetic diversity was estimated by calculating the standard diversity indices: number of haplotypes (*H*), number of segregating sites (*S*), haplotype diversity (*H*_d_), nucleotide diversity (*π*), the number of parsimony informative sites and average number of nucleotide differences (*K*). All diversity indices were calculated with DnaSP 6 software (Rozas et al., 2017). The relationships between identified haplotypes were explored through haplotype networks created in PopART 1.7 software (Leigh & Bryant, 2015), using the Templeton, Crandall and Sing (TCS) method (Clement et al., 2002). The TCS method is based on a maximum parsimony algorithm.

Three different classes of common analyses were used to infer demographic history. Tajima's *D* (Class I), which is based on the frequency of segregating nucleotide sites (Tajima, 1989) and Ramos‐Onsins and Rozas's (*R*_2_, Class I), which is calculated using the number difference between singleton count and the average number of nucleotide differences (Ramos‐Onsins & Rozas, 2002). Fu's *F* (Class II) (Fu, 1997), which uses the distribution of haplotype frequencies and, along with *R*_2_, is considered to be more sensitive than Tajima's *D* (Ramos‐Onsins & Rozas, 2002). In addition, mismatch distribution analysis based on pairwise sequence differences, and Harpending's raggedness index (*r*, Class III) (Ramos‐Onsins & Rozas, 2002) were calculated using a population growth–decline model. All demographic tests were calculated in DnaSP 6 (Rozas et al., 2017) and their significance assessed using 10,000 bootstrap replicates.

### 2.6 Population structure and connectivity analysis

To test for hierarchical population genetic differentiation, an analysis of molecular variance (AMOVA) was performed using the distribution of variation at the regional, sampling site and individual levels (Excoffier et al., 1992). Genetic connectivity between geographical regions was estimated by calculating pairwise *θ*_ST_ (fixation index among populations), for which a significance level of 0.05 was determined using 10,100 permutations (Holsinger & Weir, 2009). These analyses were conducted using Arlequin 3.5.2.

## 3 RESULTS

### 3.1 Maximum likelihood clustering of *T. abyssorum* and *T. libellula*

**FIGURE 3:**
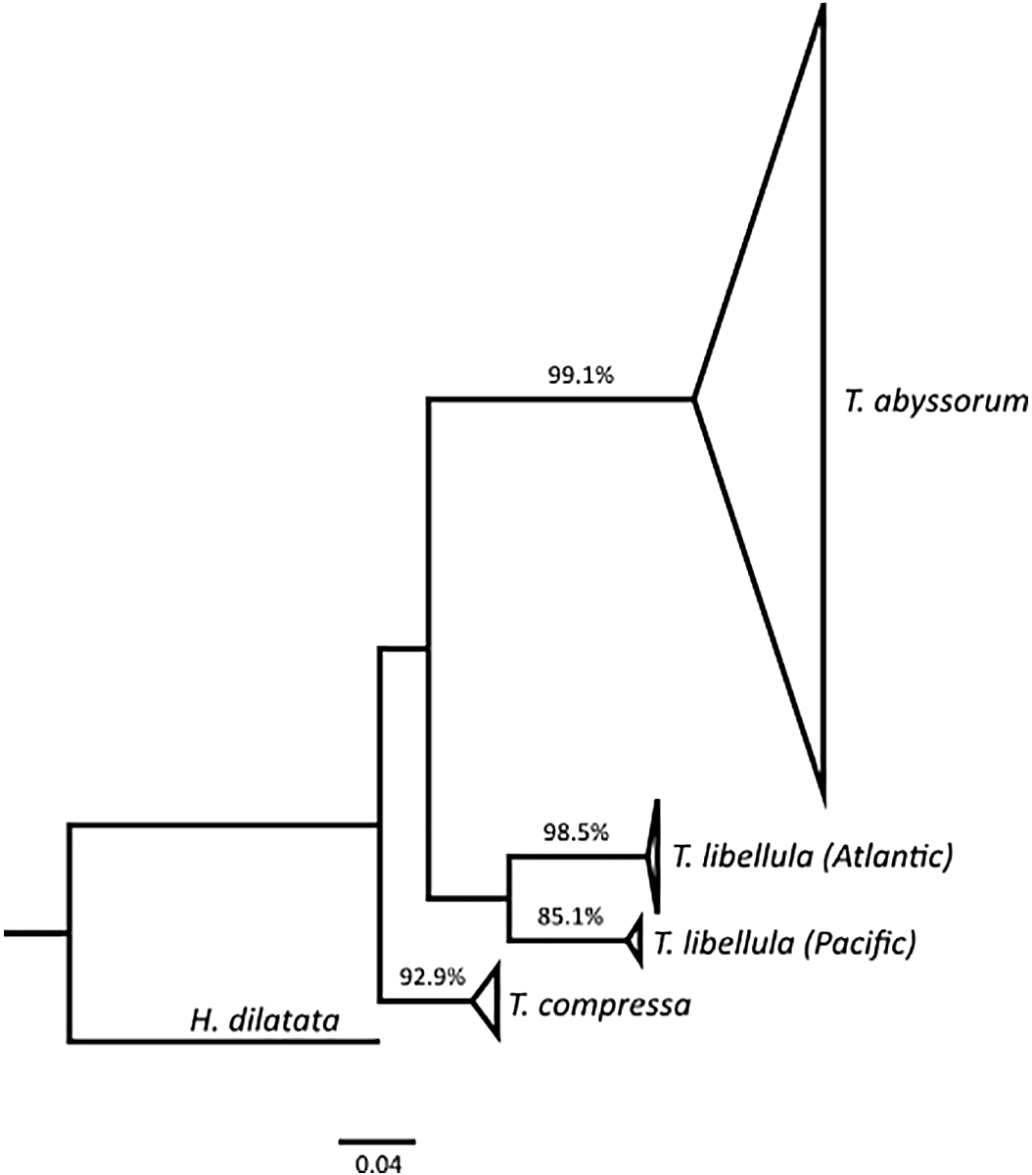
Collapsed maximum likelihood phylogenetic tree of *Themisto libellula*, *Themisto abyssorum* and *Themisto compressa*. Tree is based on COI sequences. Numbers represent statistical support for clades, tested using bootstrap analysis with 1000 replicates. Outgroup is the species *Hyperiella dilatata*.

All *Themisto* barcodes clustered in the ML tree with high bootstrap support for species‐level clusters (>85%) (Figure 3). The *T. libellula*, *T. abyssorum* and *T. compressa* specimens sampled in this study were recovered in single clusters for each species. However, the *T. libellula* GenBank sequences of individuals sampled in the Pacific Arctic waters were recovered in a highly divergent cluster, indicating the presence of a possible species complex, with another species‐level lineage morphologically assigned to *T. libellula*. The K2P genetic distances between the Atlantic *T. libellula* (sampled in this study) and the Pacific *T. libellula* sequences were similar (9.72%) to the distances between Atlantic *T. libellula* and *T. compressa* (10.0%), further indicating the presence of a species complex. Similar K2P distances of 10% were also found between other *Themisto* species in the study of Tempestini et al. (2017). The highest K2P distance observed in this study was between the *T. libellula* from the Atlantic Arctic and *T. abyssorum* (16.34%; Table 2). Comparatively high distance values were previously observed between *T. abyssorum* and *T. gaudichaudii* (Tempestini et al., 2017).

**TABLE 2:** Estimates of inter‐ and intraspecific genetic distances (above and in the diagonal respectively).

	*T. libellula* (Atlantic Arctic)	*T. libellula* (Pacific Arctic)	*T. abyssorum*	*T. compressa*
*T. libellula* (Atlantic Arctic)	**0.07%**	9.72%	16.34%	10.00%
*T. libellula* (Pacific Arctic)		**0.29%**	13.66%	11.20%
*T. abyssorum*			**1.29%**	14.84%
*T. compressa*				**0.20%**

*Note*: Analyses were conducted using the K2P model and calculated in MEGA 11. The number of sequences used were 472 and there was a total of 669 positions in this analysis. Values in bold text indicate intraspecific distances.

### 3.2 Genetic diversity of *T. libellula* and *T. abyssorum* in the Atlantic Arctic

The 250 *Themisto libellula* specimens exhibited a total of 16 polymorphic sites, 4 parsimony informative sites and 16 unique haplotypes (*H*). Genetic diversity was low with haplotype diversity, *H*_d_ = 0.360 (SD = 0.0360); nucleotide diversity, *π* = 0.0006 (SD = 0.00008); and average number of nucleotide differences, *K* = 0.42 (Table 3). Intraspecific genetic distance was the lowest among the species studied (0.07%; Table 2). In contrast, 136 polymorphic sites and 68 parsimony informative sites were identified for *T. abyssorum*, as well as 115 unique haplotypes belonging to 152 individuals. Genetic diversity indices were higher with *H*_d_ = 0.975 (SD = 0.008), *π* = 0.01218 (SD = 0.00089) and *K* = 8.02 (Table 3). Intraspecific genetic distance was higher than the other species clusters at 1.29% (Table 2).

**TABLE 3:** Genetic diversity indices and demographic analyses based on mtCOI for *Themisto libellula* and *Themisto abyssorum* obtained in this study.

	*T. libellula*	*T. abyssorum*
Diversity indices		
Sample size (*N*)	250	152
Number of haplotypes (*H*)	16	115
Polymorphic sites (*S*)	16	136
Parsimony informative sites	4	68
Haplotype diversity (*H*_d_ ± SD)	0.360 ± 0.036	0.975 ± 0.008
Nucleotide diversity (*π* ± SD)	0.00064 ± 0.00008	0.012818 ± 0.00089
Average number of nucleotide differences (*K*)	0.42	8.02
Tajima's *D*	−2.11*	−2.16*
Fu's *F*	−19.40*	−32.85*
*R*_2_	0.02	0.29*
Raggedness index (*r*)	0.176	0.0086*

Abbreviation: SD, standard deviation.

* Significant *p*‐value (<.05).

**FIGURE 4:**
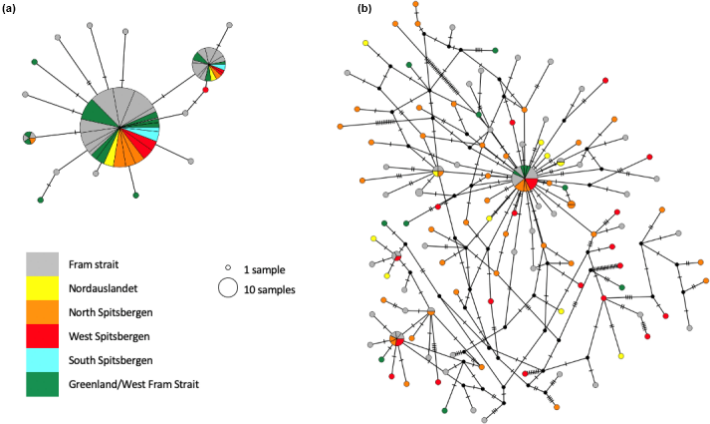
TCS haplotype networks for (a) *Themisto libellula* and (b) *Themisto abyssorum* based on mtCOI sequences. Each haplotype is represented by a circle and is coloured according to its geographical region. Circle represents haplotype frequency; hypothetical or missing haplotypes are represented by notches and mutations are represented by bars.

TCS haplotype networks based on mtCOI region illustrate contrasting diversity between the two species in the Atlantic Arctic study region (Figure 4). *T. libellula* showed two dominant haplotypes and 13 singletons. All haplotypes diverge by only one or two of mutational steps, which is reflected in the low nucleotide diversity value (*π*; Table 3). In contrast, *T. abyssorum* exhibited a large number of highly divergent haplotypes, with only one haplotype occurring in more than 10 individuals, and 107 singleton haplotypes.

The results of the Tajima's *D* and Fu's *F* tests for neutrality were significantly negative for both species (Table 3). These results indicate a recent population expansion and allow for the rejection of the null hypothesis that the sampled populations are in a state of equilibrium. Mismatch distribution analysis for *T. libellula* showed a skewed unimodal distribution and a nonsignificant *r* index, which further supports a recent population expansion. However, *T. abyssorum* had a multimodal (‘ragged’) distribution and significant *r* index, indicating either a stable population size or the presence of multiple lineages (Jenkins et al., 2018).

### 3.3 Population structure and connectivity

**FIGURE 5:**
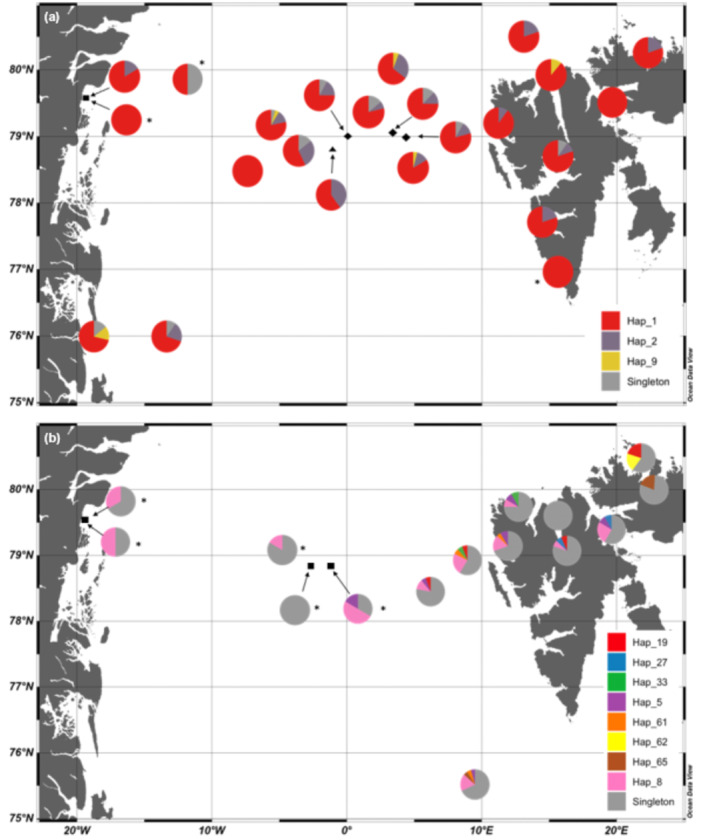
Relative frequency of *Themisto libellula* (a) and *Themisto abyssorum* (b) haplotypes at each sampling station. Colour indicates individual haplotypes. * indicates sites where *N* < 4.

The data revealed a lack of genetic structure for both species in all the sampled geographical regions. The TCS haplotype networks for *T. libellula* showed that the two most common haplotypes (Hap 1 = 197 and Hap 2 = 35 individuals; Figure 5) were present in all six of the main geographical regions sampled (Figure 4). The third most common haplotype (Hap 9 = 5) was present at three out of the six regions (Figure 5). This lack of spatial genetic structure was further underlined by a single haplotype (Hap 1) dominating the haplotype frequency at each sampling station, except one, for *T. libellula* (Figure 5). The haplotype network for *T.abyssorum* showed that the dominant haplotype (Hap 8 = 23) was present at four out of five of the regions sampled (no *T. abyssorum* sequences were obtained from South Spitsbergen) and is present at all except the two Nordaustlandet stations. Every station, except for one Fram Strait station is dominated by singletons (Figure 5).

The AMOVA of spatial patterns of genetic variation within and among sampled populations showed no statistical significance of any of the variance components (among regions, among stations or among individuals). Based on these results, neither species exhibited significant geographical structure among the sampled populations (Table S1). Pairwise *θ*_ST_ comparisons between the geographical regions showed no significant differences between pairs of regions, further indicating a lack of genetic structure and high levels of connectivity between the Atlantic and Arctic regions in both *T. libellula* and *T. abyssorum* (Table S2).

## 4 DISCUSSION

Two abundant pelagic amphipod species were analysed for genetic diversity, demographic history, and patterns of spatial genetic structure and connectivity, using variation at the mitochondrial COI region. Overall, the levels of mtCOI diversity were strikingly different: low in *T. libellula* and high in *T. abyssorum*. No evidence of spatial genetic structure but high levels of connectivity were found in both species, across the regions sampled in the Atlantic sector of the Arctic. Indications of demographic expansions were identified, with stronger signals detected in *T. libellula*. When comparing these results to sequences from individuals morphologically assigned to *T. libellula* from the Pacific Arctic, clear genetic differences were observed between the lineages, likely indicating a separate species.

### 4.1 Genetic diversity within *T. libellula* and *T. abyssorum*

We found that *T. libellula* had low nucleotide and haplotypic diversity with only weakly divergent haplotypes. The majority of the individuals grouped into two dominant and potentially ancestral haplotypes, connected to multiple haplotypes occurring at low frequencies (Figure 4). Such star‐like haplotype networks are often indicative of a bottleneck event followed by population expansions (e.g. Allcock & Strugnell, 2012; Jenkins et al., 2018; Strasser & Barber, 2009). Similarly low diversity indices were reported in a recent study by Tempestini et al. (2020) on *T. libellula* in the Pacific Arctic, based on both mtCOI and nuclear genomic markers. They found low haplotype diversity values in populations in the Chukchi Sea, the Bering Sea and the Gulf of Alaska. However, when combined in a ML‐tree with the sequences obtained herein, the Pacific sequences clustered in a divergent clade. This indicates that either the *T. libellula* in Pacific waters are a separate species or that the *T. libellula* in Arctic waters should be regarded as a species complex. The latter is supported by our K2P divergence estimates and has previously been suggested by Tempestini et al. (2017). Whether these species‐level lineages also differ in morphology cannot be confirmed with the present data and should be the focus of future studies.

Haplotypic diversity in *T. abyssorum* was more than two‐fold higher, and the number of unique haplotypes was close to 10 times that found in *T. libellula*, despite a smaller sample size. Nucleotide diversity was also more than one magnitude higher than that of *T. libellula*, alluding to high genetic distances between haplotypes. A higher haplotype diversity was also observed in temperate *Pseudocalanus* copepods when compared to an Arctic congeneric species (Questel et al., 2016). The high genetic diversity in *T. abyssorum* is also evident in the haplotype network, which is a ‘diffuse’ shape with numerous mutations separating the many haplotypes. This network shape has been found in other species with deep‐sea distributions, including the benthic shrimp, *Nematocarcinus lanceopes* (Raupach et al., 2010) and the squat lobsters, *Munida endeaviurae* and *Munida gracilis* (Yan et al., 2020).

The highly divergent patterns of genetic diversity found in *T. libellula* versus *T. abyssorum* in the Atlantic sector of the Arctic, combined with the findings of a species complex in *T. libellula* on the pan‐Arctic scale, may have alternative explanations. Evolutionary bottlenecks occurred for many marine species in the Arctic during historic glacial cycles, including the LGM (approximately 20,000 years ago) (Csapó et al., 2021; Eldevik et al., 2014). During these periods, the expansion of both continental and maritime ice sheets covered the continental shelves (e.g. Barents and Kara seas; Batchelor et al., 2019; Ehlers & Gibbard, 2008), as well as the central Arctic basins (Jakobsson et al., 2016). Thick, perennial sea‐ice cover in other areas (e.g. Fram Strait, Müller et al., 2009), prevented algal production during spring and summer, leading to severely limited viable habitats for pelagic species. Glacial refugia for pelagic species may have persisted in the form of polynyas and large open‐water corridors between ice sheet edges, or species may have migrated southwards to more ice‐free waters. This extreme reduction of available habitat, combined with periods of isolation in refugia, caused declines in population size and genetic diversity across many marine Arctic taxa (Hardy et al., 2011; Hewitt, 2004). Subsequent deglaciation was followed by rapid recolonization, causing founder effects in species of which few individuals, or individuals with low genetic variation, were able to recolonize the area (Hardy et al., 2011; Shimizu et al., 2018). The signatures of the last glacial cycle of the contemporary genetic diversity thus depend on the size and number of refugia, the effective population size and the standing genetic variation of the survivors (Hardy et al., 2011; Hewitt, 2000).

Based on our results, *T. libellula* may have been isolated in different glacial refugia during the LGM, where the isolated populations diverged from each other, resulting in an increase in local genetic diversity (the vicariant effect). Once the populations were able to recolonize the Arctic, secondary contact between the populations may then have resulted in an overall increase in intraspecific diversity or have caused speciation in the shape of species complexes (Hardy et al., 2011). This could explain the occurrence of distinct species‐level *T. libellula* lineages in different Arctic regions. The results herein also provide strong evidence of demographic expansion in *T. libellula*, supporting the scenario of utilization of different refugia followed by secondary contact and rapid population expansion after the LGM. Generally, similar genetic signatures of the LGM have been identified in other zooplankton taxa in the Arctic including the pteropod *L. helicina* (Shimizu et al., 2018; Sromek et al., 2015), the copepod *Pseudocalanus moultoni* (Aarbakke et al., 2014) as well as in fish species, such as the circum‐Arctic capelin (*Mallotus villosus*; Dodson et al., 2007). However it remains unclear whether the low genetic diversity in certain Arctic species is caused entirely by limited former refugia, colonization following deglaciation and consequent founder effects (Shimizu et al., 2018). Mutations that have occurred since may explain the presence of rarer haplotypes (Lasota et al., 2016), however, the time since the LGM is likely insufficient for many mutations to have accumulated in *T. libellula* (Grant et al., 2011). An alternative explanation that cannot be eliminated based on the current dataset is that of a mitochondrial selective sweep reducing genetic variability on the mtCOI gene (e.g. Hurtado et al., 2004; Jenkins et al., 2018; Lasota et al., 2016; Strasser & Barber, 2009). This hypothesis could however be ruled out when carrying out a multigene study, as it would be unlikely that selective sweeps occur in several unlinked mitochondrial and nuclear markers (Strasser & Barber, 2009).

Meanwhile, *T. abyssorum*, being a subarctic boreal‐Atlantic species distributed across both the Atlantic and Arctic oceans (distribution maps in Havermans, Auel, et al., 2019), may have experienced less demographic impact from previous glaciation events than the strictly polar *T. libellula* due to milder climate conditions, large refugia remained in Europe during the LGM (Maggs et al., 2008; Wares & Cunningham, 2001). The ability of *T. abyssorum* to thrive in Atlantic waters (<50 m; Dalpadado, 2002) in the subarctic and Atlantic Ocean may have prevented this species from experiencing a major habitat loss followed by population declines. It is therefore possible that a larger, stable and interconnected metapopulation was maintained across the Atlantic. Our results showed evidence of post‐LGM demographic population expansion in *T. abyssorum* and also stable population size composed of multiple lineages. Although multiple lineages are not necessarily reflected through the presence of multiple dominant haplotypes in the haplotype network (Figure 4), the high number of mutations between many of the haplotypes may support this result (Jenkins et al., 2018). A stable population of *T. abyssorum* would also be in line with the aforementioned LGM scenario. The discrepencies in the demographic output may represent interesting information about the population history of the species and should be further investigated, using more sampling regions and multiple genetic markers.

### 4.2 Population structure and connectivity

The results herein did not reveal any significant genetic differentiation among sampling locations for either of the two species, as clearly illustrated in the haplotype networks, haplotype distribution maps and subsequent statistical analysis (AMOVA, *θ*_ST_). Dominant haplotypes were present across the sampling area for both species and all pairwise *θ*_ST_ values were low and insignificant between the major geographical regions. Collectively, we interpret this as an indication of high levels of connectivity and genetic homogeneity for both species, within the Atlantic–Arctic region. Weak or non‐existent population structure is common among marine species with pelagic life stages, such as *Themisto* amphipods, due to the high potential for dispersal and gene flow through ocean currents (Hardy et al., 2011). Similar high levels of population connectivity have been demonstrated in other key Arctic zooplankton species including *Calanus* copepods (Weydmann et al., 2014, 2018), chaetognaths (DeHart et al., 2020), as well as in intertidal amphipods (Grabowski et al., 2019), and for multiple polychaete and echinoderm species with planktonic life stages (Hardy et al., 2011). The high gene flow between the studied populations is likely heavily mediated by the current regimes in the Fram Strait and around the Svalbard Archipelago. The WSC carries water northward as well as circulating it across the Fram Strait, the ECG carries water southward and the SCC carries polar water around the southern tip of Spitsbergen and northward again. This lack of population structure and high levels of connectivity also support the evidence of demographic expansion discussed in the previous section, but to which extent remains to be investigated. Such a study would also benefit from the inclusion of more mtDNA gene regions and a dense coverage of nuclear DNA markers, to allow identification of the unique traces of demographic history, dispersal and migration. Furthermore, it would allow for insight into the impacts of changes in effective population size on diversity, and how adaptive traits influence population structure (Grant, 2015; Madoui et al., 2017; Peijnenburg & Goetze, 2013).

### 4.3 Future implications for the rapidly changing Atlantic–Arctic

Shifts in distribution linked to environmental changes have already been reported in *Themisto* species in the Arctic and its adjacent seas. Abundances of *T. libellula* in the Barents Sea and the Fram Strait have been reportedly decreasing in recent years (CAFF, 2017). Dalpadado et al. (2012) found significant correlation between decreases in *T. libellula* (and other Arctic zooplankton species) and warming trends across the Barents Sea over a period of 25 years. Increases in presence and abundances of *T. libellula* were observed in colder years in the southern Bering Sea, and found to disappear in warmer years (Pinchuk et al., 2013). Mass occurrences of *T. libellula* have been reported in northern parts of the Bering Sea during periods of cold‐water inflow, but decreased with subsequent warming (Volkov, 2012). Although these distribution changes are not all poleward, the authors identify temperature changes as the main driver behind species presence and abundance, with *T. libellula* populations closely following the movements of colder waters.

An opposite effect of temperature has been observed for *T. abyssorum* in the Fram Strait, where a sediment trap time series analysis revealed increased abundance with increasing warming events (CAFF, 2017; Kraft et al., 2013). Presence of the invasive boreal *T. compressa* has been reported in the Fram Strait since 2004, with proof of established reproductive events in 2011 (Kraft et al., 2013; Schröter et al., 2019). Other Arctic pelagic zooplankton species, including krill species (Buchholz et al., 2010) and *Calanus* copepods (Weydmann et al., 2014), have shown community and distribution changes as a result of Atlantification around the Svalbard Archipelago and in Fram Strait. These changes in distribution and abundance associated with the changing environmental conditions in the Arctic are evidence that zooplankton communities are already in transition to a warmer Arctic dominated by boreal species (Csapó et al., 2021).

A combination of factors, including low genetic diversity at the COI gene, long life cycle (Havermans, Hagen, et al., 2019), cold‐water affinity and its potential associated adaptions (Pinchuk et al., 2013), and reliance on the cryo‐pelagic pathway (Auel et al., 2002; Kohlbach et al., 2016), may well lead to *T. libellula* being negatively impacted by the Atlantification of the Arctic. This could result in loss of intraspecific diversity, local extinctions and further poleward distribution contractions with unknown effects on the food web. In contrast, *T. abyssorum* could benefit from an increasing Atlantification, as it displays an Atlantic affinity and a shorter life cycle (Havermans, Auel, et al., 2019). Moreover, the high standing genetic variation observed in *T. abyssorum*, if confirmed at the genome level, can be an important source of swift adaption to selective pressures, whereas species with a low standing genetic variation are potentially more susceptible to rapid environmental changes (Thompson et al., 2019). The possible replacement of the large, nutritionally rich *T. libellula* with the smaller *T. abyssorum* and *T. compressa* is likely to negatively impact predators at the higher trophic levels. Arctic species that specialize in feeding on *T. libellula*, for example the little auk and polar cod, will have to adapt to a new prey spectrum consisting of smaller and less energy‐rich boreal species such as *T. abyssorum* (Dalpadado et al., 2012; Kraft et al., 2013).

In conclusion, this study shows for the first time, contrasting molecular diversity between two congeneric species of pelagic amphipods. It contributes to a better understanding of the evolutionary processes driving molecular diversity and the adaptive potential of two ecologically important species in a changing Arctic. These results emphasize the need for further analysis of the molecular biogeography and phylogeny of key zooplankton species in the Arctic Ocean. A better understanding of population connectivity, physiology, adaptive potential and trophic ecology is crucial for formulating accurate predictions of how future zooplankton communities, species interactions and food‐web structure will materialize in the Arctic and its marginal seas as a result of climate change.

## AUTHOR CONTRIBUTIONS

**Ayla Murray:** Conceptualization (supporting); formal analysis (lead); investigation (lead); writing – original draft (lead); writing – review and editing (equal). **Kim Præbel:** Funding acquisition (supporting); investigation (supporting); writing – review and editing (supporting). **Andrea Desiderato:** Formal analysis (supporting); validation (supporting); writing – review and editing (supporting). **Holger Auel:** Funding acquisition (supporting); investigation (supporting); writing – review and editing (supporting). **Charlotte Havermans:** Conceptualization (lead); funding acquisition (lead); investigation (supporting); supervision (lead); writing – original draft (supporting); writing – review and editing (equal).

## CONFLICT OF INTEREST STATEMENT

None declared.

## Supporting information


Figure S1

Figure S2

Table S1

## Data Availability

Sequence data and sampling locations of all analysed specimens are publicly available on the BOLD depository under the project name ‘ARCTH’ and made available on GenBank (Accession numbers: OR210933–OR211370).
