# Supplementary figures and images for: Phylogeography and molecular diversity of two highly abundant Themisto amphipod species in a rapidly changing Arctic Ocean
Source: Ecol Evol. 2023 Jul 30;13(8):e10359. doi: 10.1002/ece3.10359 (PMC10387590; doi:10.1002/ece3.10359)

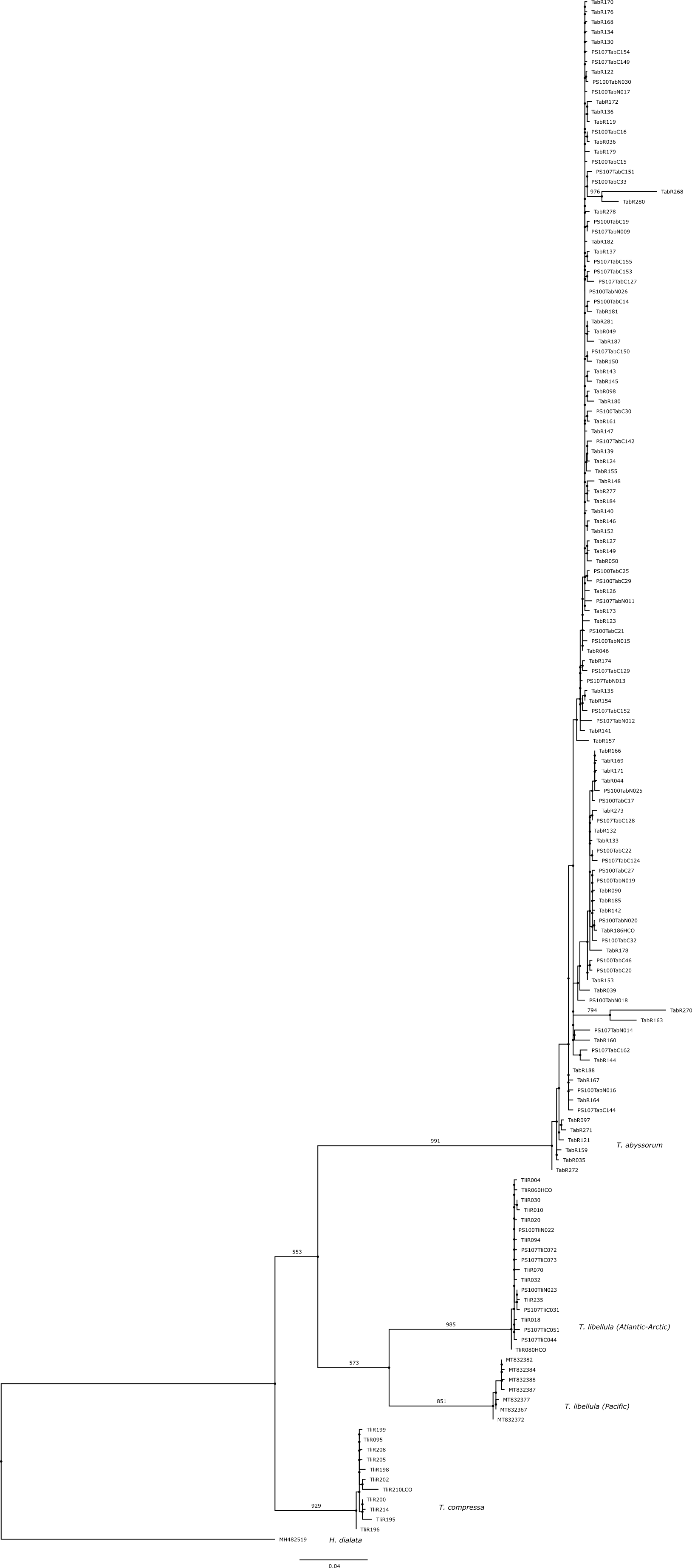

Supplement: Supplementary file 1 — Figure S1 [file ECE3-13-e10359-s003.png]

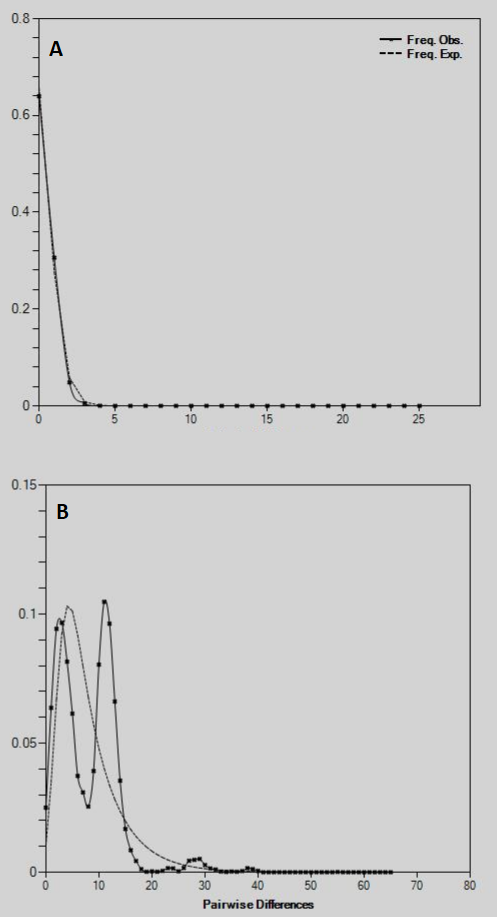

Supplement: Supplementary file 2 — Figure S2 [file ECE3-13-e10359-s001.png]
