# Supplementary material for: Phylogeography and molecular diversity of two highly abundant Themisto amphipod species in a rapidly changing Arctic Ocean
Source: Ecol Evol. 2023 Jul 30;13(8):e10359. doi: 10.1002/ece3.10359 (PMC10387590; doi:10.1002/ece3.10359)
Supplement: Supplementary file 3 — Table S1 [file ECE3-13-e10359-s002.docx]

# **Appendix S1**

**Supporting Information**

File name: Appendix S1

Description:

- S1 Table 1: The results of spatial population genetic structure using the Analysis of Molecular Variance (AMOVA) based on mtCOI.
- S1 Table 2: Results of pairwise θ_ST_ values based on mtCOI for genetic connectivity between major geographic regions.
- S1 Figure 1: Extended Maximum Likelihood phylogenetic tree of *T. libellula*, *T. abyssorum* and *T. compressa*. Tree is based on COI sequences. Numbers represent statistical support for clades, tested using bootstrap analysis with 1,000 replicates. Outgroup is the species *H. dilatata*.
- S1 Figure 2: Mismatch distribution plots for (A) *T. libellula* and (B) *T. abyssorum*, calculated in DnaSP 6 software.

| **S1. Table 1**. The results of spatial population genetic structure using the Analysis of Molecular Variance (AMOVA) based on mtCOI. Significance tests based on 10100 permutations. Abbreviations: *df*= degrees of freedom, SS= Sums of Squares, F= fixation index. | | | | | | |
| --- | --- | --- | --- | --- | --- | --- |
| *Themisto libellula* | |  |  |  |  |  |
| **Source of variation** | ***df*** | **SS** | **Variance Component** | ***%* variation** | **F** | ***p-value*** |
| Between regions | 5 | 0.614 | -0.00042 | -0.23 | -0.02596 | 0.55851 |
| Between stations | 17 | 2.374 | -0.00425 | -2.36 | -0.02357 | 0.82733 |
| Between individuals | 227 | 41.867 | 0.18444 | 102.60 | -0.00233 | 0.88782 |
| Total | 249 | 44.856 |  | 100 | - | - |
| *Themisto abyssorum* | |  |  |  |  |  |
| **Source of variation** | ***df*** | **SS** | **Variance Component** | ***%* variation** | **F** | ***p-value*** |
| Between regions | 4 | 1.989 | 0.00263 | 0.54 | 0.00540 | 0.09485 |
| Between stations | 11 | 4.904 | -0.00550 | -1.13 | -0.01134 | 0.90584 |
| Between individuals | 136 | 66.719 | 0.49058 | 100.59 | -0.00588 | 0.91673 |
| Total | 151 | 73.612 | 0.48771 | 100 | - | - |

| **S1. Table 2.** Results of pairwise θ_ST_ values based on mtCOI for genetic connectivity between major geographic regions. No significant values were detected. All negative values are taken to be zero. | | | | | | |
| --- | --- | --- | --- | --- | --- | --- |
| *T. libellula* | | | | | | |
|  | Fram Strait | Greenland Shelf/ West Fram Strait | Nordauslandet | North Spitsbergen | West Spitsbergen | South Spitsbergen |
| Fram Strait | 0 |  |  |  |  |  |
| Greenland Shelf/ West Fram Strait | -0.00421 | 0 |  |  |  |  |
| Nordauslandet | -0.05043 | -0.04769 | 0 |  |  |  |
| North Spitsbergen | 0.023 | -0.00397 | -0.00857 | 0 |  |  |
| West Spitsbergen | -0.00515 | -0.02249 | -0.05296 | -0.0282 | 0 |  |
| South Spitsbergen | -0.02926 | -0.03929 | -0.08893 | -0.03214 | -0.05686 | 0 |
| *T. abyssorum* |  |  |  |  |  |  |
|  | Fram Strait | Greenland Shelf/ West Fram Strait | Nordauslandet | North Spitsbergen | West Spitsbergen | - |
| Fram Strait | 0 |  |  |  |  |  |
| Greenland Shelf/ West Fram Strait | -0.01985 | 0 |  |  |  |  |
| Nordauslandet | -0.03077 | -0.03182 | 0 |  |  |  |
| North Spitsbergen | -0.00099 | -0.01505 | -0.01953 | 0 |  |  |
| West Spitsbergen | -0.00808 | -0.02079 | -0.0403 | 0.00697 | 0 |  |
